# Youth mental health resilience during the COVID-19 pandemic: A critical review

**DOI:** 10.1192/j.eurpsy.2024.685

**Published:** 2024-08-27

**Authors:** S. Irnat, S. Boukhorb, S. Hmimou, M. Anssoufouddine, A. Soulaymani, M. Abdalli Mari, A. Mokhtari, H. Hami

**Affiliations:** ^1^Laboratory of Biology and Health, Faculty of Sciences, Ibn Tofail University, Kenitra, Morocco; ^2^Medical Service, Regional Hospital Center of Anjouan, Mutsamudu, Comoros

## Abstract

**Introduction:**

The COVID-19 pandemic has had a profound impact on mental health worldwide because of complex societal disruptions and neuropsychiatric consequences stemming from SARS-CoV-2 infection. All age groups have been affected by this pandemic, with particular focus on the vulnerabilities faced by children and adolescents who have experienced multiple stressors. These stressors involve various emotional, physiological, and behavioral challenges stemming from different factors, such as mandatory social distancing due to school closures, increased parental stress caused by the incessant spread of the pandemic, severe trauma from losing family members, a surge in cyberbullying linked to higher online activity, and a worrying rise in unreported incidents of child abuse. Empirical reports document an increase in suicidal tendencies and suicide attempts among adolescents during this crisis.

**Objectives:**

This study conducted a comprehensive review of existing literature focused on the mental health of individuals aged 0-24 years in both pre-pandemic and pandemic eras. This study conducted comparative analyses to identify significant changes.

**Methods:**

Adhering strictly to the PRISMA guidelines, we conducted comprehensive searches on Google Scholar and PubMed to identify peer-reviewed articles published in English.

**Results:**

Most studies revealed deteriorating mental health conditions among adolescents and young adults following pandemic onset. These conditions were characterized by high rates of depression, anxiety, and psychological distress. Furthermore, several studies have identified a notable increase in negative emotions and heightened feelings of loneliness. Primary school children experienced a decline in attention span, emotional regulation, hyperactivity, and enthusiasm for academic engagement.

**Conclusions:**

Based on the analysis of data from both the pre-pandemic and pandemic periods, it is evident that the COVID-19 pandemic had a detrimental impact on the mental well-being of children and young individuals. Therefore, it is crucial to identify the risk factors and protective measures linked with pandemics to enhance mental health resilience and better equip societies to cope with future health emergencies and other crises.

**Disclosure of Interest:**

None Declared

